# Suprasternal Schwannoma: A Rare Site of Presentation

**DOI:** 10.7759/cureus.28569

**Published:** 2022-08-30

**Authors:** Mithula Murali, Shraddha Jain, Chandra Veer Singh, Ajinkya Sandbhor, Vaidehi Hande

**Affiliations:** 1 Otolaryngology - Head and Neck Surgery, Jawaharlal Nehru Medical College, Datta Meghe Institute of Medical Sciences, Wardha, IND

**Keywords:** schwannoma, schwann cells, neurogenic tumour, fascicular sign, suprasternal

## Abstract

Schwannomas are benign neurogenic tumours, arising from Schwann cells in the nerve. These tumours are a rare occurrence; 25% are reported in the head and neck region in adults, are rare in the paediatric population, and have a male preponderance. Radiologic investigations can assist in the diagnosis, and surgical excision is the treatment of choice. We report a case of suprasternal schwannoma, which is a rare site of occurrence in the neck, in a 14-year-old male child. Complete resection of the tumour was performed. The patient was free of recurrence with no symptoms at six-month follow-up.

## Introduction

Schwannomas are benign neurogenic tumours that arise from the Schwann cells in the nerve [1-4]. They are more commonly located in the thorax, at the spinal nerve roots, and in intercostal nerves, brachial plexus, phrenic nerve, sympathetic chain, and extremities [2]. Twenty-five to forty-five percent of extracranial schwannoma is present in the head and neck region [2]. The vagus nerve is the most common site. [1,5]. It is common in the adult population [1,3]. Symptoms may differ according to the location of the tumour and also compression on the adjacent structures. Imaging such as MRI needs to be done to reach an early diagnosis, thus preventing complications [2]. Literature shows that the sensitivity of fine needle aspiration cytology in the diagnosis of schwannoma was 71.4% [5]. These tumours, being well encapsulated, are resistant to radiotherapy. They are usually unilateral and slow-growing tumours [6]. A study showed that the nerve of origin from major nerve could not be identified in 17% of cases [7]. Surgical excision is the treatment modality in cases of rapidly growing or symptomatic tumours [2]. We present a case of suprasternal schwannoma, a rare site of occurrence in the neck, in which the nerve of origin could not be identified, of a 14-year-old male child. Complete surgical resection of the tumour was done without any morbidities. This is probably the first case being reported of a schwannoma in a suprasternal location.

## Case presentation

**Figure 1:**
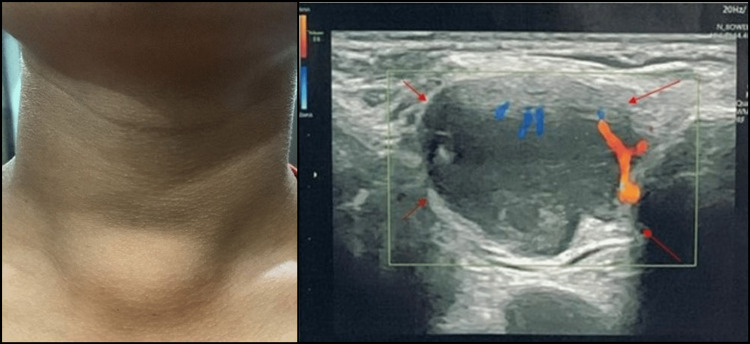
Midline neck swelling and ultrasonography neck: red arrows suggested a hypoechoic lesion of approximately 18 x 33 x 24 mm in the suprasternal region

 A 14-year-old male child presented to the otolaryngology outpatient department with swelling over the lower aspect of the neck in the midline and mild dull aching pain over the swelling for six months. He did not complain of weakness, abnormal sensation, or numbness in his hands. Physical examination revealed a smooth-surfaced and painless, firm swelling of about 4 x 3 cm in the midline of the neck at the suprasternal region with no retrosternal extension and no fixity to underlying structure or skin. No other neck masses like cervical lymph nodes were palpable (Figure 1). Ultrasonography suggested a hypoechoic lesion of approximately 18 x 33 x 24 mm in the suprasternal region (Figure 1).

**Figure 2:**
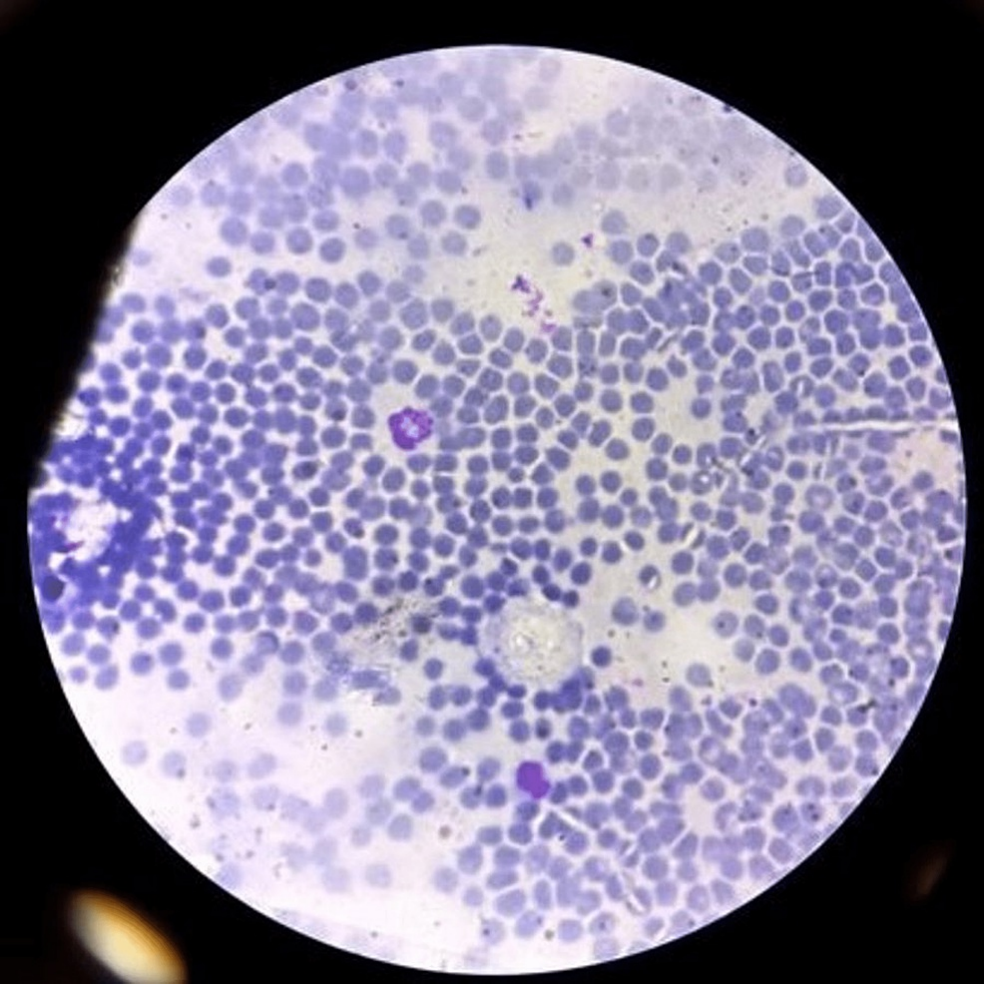
Ultrasonography-guided fine needle aspiration cytology (FNAC) with immuno-stain suggestive of schwannoma

Ultrasonography-guided fine needle aspiration cytology (FNAC) with immuno-stain was performed; smear shows fragments of spindle nuclear cells placed parallelly and in one or two fragments, appear to be placed whorled. The cells carry bipolar tapering wavy nuclei with a little hyperchromasia without significant pleomorphism. The cytoplasm appears to be merged in the background, with few isolated spindle nuclei with cytoplasmic viscous and scattered lymphocytes. These are features suggestive of schwannoma (Figure 2).

**Figure 3:**
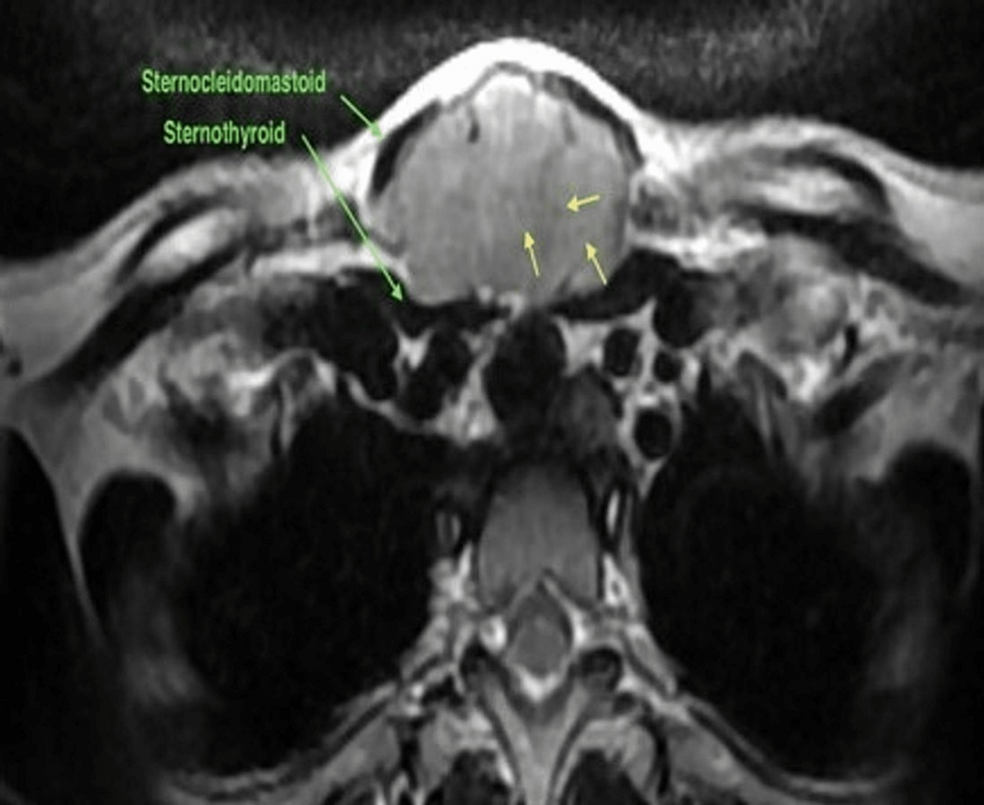
MRI neck (plain and contrast) suggestive of peripheral nerve sheath tumour. Schwannoma in the suprasternal region with yellow arrows showing fascicular sign

MRI neck with contrast showed well defined ovoid intensely enhancing mass noted in the midline lower neck suprasternal region of size 5 x 3.2 x 2.7 cm, insinuating between the lower end of sternocleidomastoid and strap muscles of the neck bilaterally with characteristic fascicular sign, showing structures arranged in a ring with peripheral hyperintensity, suggestive of peripheral nerve sheath tumour, most likely a schwannoma (Figure 3).

**Figure 4:**
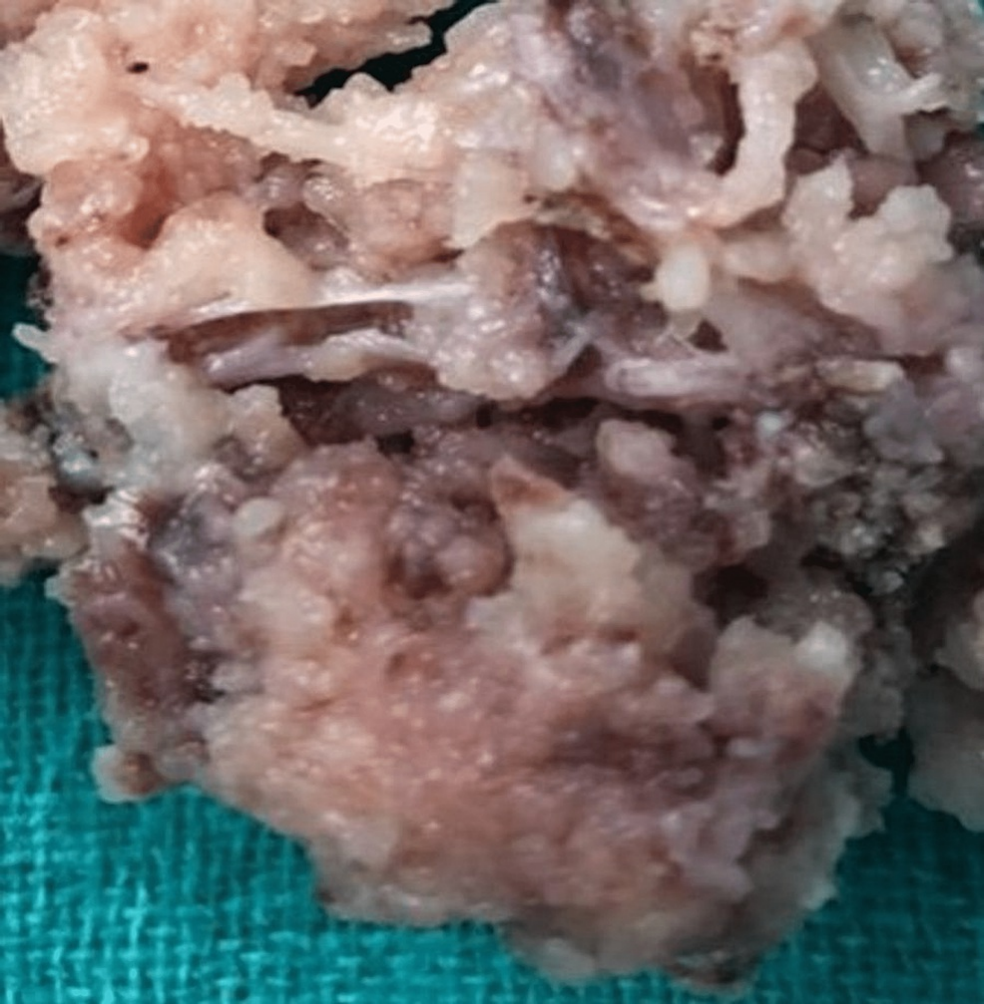
Postoperative specimen

Surgical resection of the mass was performed. The tumour was freed from the surrounding structure including sternocleidomastoid muscles, bilateral sternoclavicular joints, and suprasternal notch. Tumour appeared to be vascular. Complete excision by piecemeal achieved. The nerve of origin could not be identified (Figure 4). Histopathological examination revealed a mesenchymal tumour (neural origin). Immunohistochemistry (IHC) done S-100 protein positive for schwannoma. The patient did not have any symptoms or complications during the post-operative period and after a six-month follow-up. 

## Discussion

Schwannomas are rare, with the incidence being only 5% of all benign soft tissue tumours [2]. Twenty-five to forty-five percent of extracranial schwannomas are present in the head and neck region [2]. They typically present as painless, asymptomatic neck mass and are rare in the pediatric population [4]. Literature shows that the nerve of origin could not be identified in 4% of cases [3]. We could not identify the nerve of origin in our patient. The diagnosis is based on clinical suspicion and confirmed using the histopathological examination [4].

Schwannomas are usually benign, and resection of tumours can cause significant postoperative morbidity. It is therefore important to reach an accurate diagnosis preoperatively so that permanent neurological sequelae can be predicted and the patient counselled accordingly. Moreover, schwannomas can present in multiple sites in the head and neck region and mimic a number of other pathologies [2]. The patient being a child, the use of non-irradiating imaging modalities such as ultrasound was preferred, which enabled the exclusion of congenital pathologies like lymphangiomas [8]. The differential diagnosis of midline neck masses includes thyroglossal cyst [9]. Hence, adequate preoperative imaging should be carried out to obtain information about the tumour and come to a probable accurate diagnosis. Schwannomas have characteristic features on ultrasound scanning, CT, and MRI which may help differentiate them from other tumours such as paragangliomas [2]. MRI is considered to be superior to CT for evaluating these tumours. On non-contrast CT, schwannomas are hypodense with muscle. Contrast CT will show either homogeneously solid or heterogeneously patchy enhancement. MRI shows a well-circumscribed homogeneous mass with a relatively homogeneous low signal intensity on T1 weighting and high signal intensity, and fascicular sign (which is multiple small ring-like structures with peripheral hyperintensity) on T2 weighting [4]. Various studies have reported sensitivity of FNAC from six to twenty percent [5]. But we feel FNAC is a simple, minimally invasive, and cost-effective investigation for the diagnosis of schwannoma. Also, we feel that MRI is the most probable and best diagnostic modality for detecting schwannoma.

Surgical resection is usually the treatment of choice but in most cases, it is challenging to preserve nerve function. Because of neurological deficits associated with resection of these tumours, a conservative surgical approach such as enucleation of the tumour is preferred by some surgeons rather than complete resection from the nerve. Because of this reason, before surgery, some surgeons have advocated waiting and watching until the tumour causes significant symptoms [2]. Schwannoma is a slow-growing tumour, but in our case the patient was asymptomatic, with a swelling, which rapidly progressed to a size of 4 x 3 cm in a six-month duration, therefore we decided on surgical resection of the tumour.

## Conclusions

Head and neck schwannoma is generally a unilateral and slow-growing tumour, occurring in adults. A schwannomas is usually asymptomatic, because of which, it is considered as a difficult entity to diagnose. FNAC is helpful in accurate diagnosis, but the gold standard for diagnosis is histopathological examination. Treatment is surgical depending on the rate of growth, location of the tumour, and nerve of origin. This study aims to describe a case of a suprasternal schwannoma, found at a rare site in the head and neck region, in the pediatric population, in which nerve of origin could not be identified, with excellent surgical outcomes.,
